# Group B Streptococcal Toxic Shock Syndrome and *covR/S* Mutations Revisited

**DOI:** 10.3201/eid2301.161063

**Published:** 2017-01

**Authors:** Parham Sendi, Muad Abd el Hay, Claudia M. Brandt, Barbara Spellerberg

**Affiliations:** University of Bern, Bern, Switzerland (P. Sendi);; University of Ulm, Ulm, Germany (M. Abd el Hay, B. Spellerberg);; Johann Wolfgang Goethe University, Frankfurt, Germany (C. Brandt)

**Keywords:** toxic shock syndrome, group B Streptococcus, two-component system, bacteria, streptococci, streptococcal toxic shock syndrome, STSS

## Abstract

Gene mutations in the virulence regulator CovR/S of group A *Streptococcus* play a substantial role in the pathogenesis of streptococcal toxic shock syndrome. We screened 25 group B *Streptococcus* (GBS) isolates obtained from patients with streptococcal toxic shock syndrome and found only 1 GBS clone harboring this kind of mutation.

Streptococcal toxic shock syndrome (STSS) is typically caused by *Streptococcus pyogenes* (group A *Streptococcus* [GAS]) (*1*). Major investigations on host-pathogen interactions have been performed to determine why some persons experience uncomplicated pharyngitis, but STSS develops in others. On a molecular level, mutations in *covS* (a sensor gene of the major virulence regulator CovR/S) have been frequently associated with invasive GAS disease (*2*). In 2009, we reported a case of STSS caused by *S. agalactiae* (group B *Streptococcus* [GBS]) and *covS* mutation (*3*). Here, we reassess those findings in a larger collection of GBS isolates causing STSS.

We tested 26 GBS isolates from 25 patients (22 adults, 3 children) (Table) that were pooled from 3 countries; the United States (22 strains collected 2004–2005), Germany (1 strain, 2006), and Switzerland (2 strains, 2005). For 1 of the 2 case-patients from Switzerland, 2 isolates (same clone) were available for mutation analyses (patient 23 [*4*]). The isolate from our previously published case report (Sweden, 2005 [*3*]) served as a control strain for molecular analyses; the corresponding case-patient was included in the demographic analyses (i.e., 26 patients: 23 adults, 3 children). The median age of the included adult patients was 59 years (interquartile range 45.5–68 years); mortality rate was 35% (8/23). The ages of the 3 children were 0, 30, and 60 days (1 death).

**Table:** GBS isolates collected from patients with STSS, showing results of capsular and multilocus sequence typing

Patient no.	Isolate no.	Patient age	Outcome	Capsular serotype	Sequence type	covR/S mutation
1	BSU286	65 y	Died	II	22	–
2	BSU287	71 y	Survived	Ia	23	–
3	BSU288	46 y	Died	V	1	–
4	BSU289	46 y	Survived	IV	397	–
5	BSU290	43 y	Survived	V	1	–
6	BSU291	35 y	Died	III	19	–
7	BSU292	76 y	Survived	V	1	–
8	BSU293	48 y	Survived	Ia	23	–
9	BSU294	61 y	Died	V	1	–
10	BSU295	45 y	Survived	V	1	–
11	BSU296	63 y	Died	Ib	8	–
12	BSU297	0 d	Survived	Ia	23	–
13	BSU298	70 y	Survived	V	1	–
14	BSU299	60 y	Survived	V	1	–
15	BSU300	73 y	Survived	V	1	–
16	BSU301	66 y	Died	III	17	–
17	BSU302	2 mo	Survived	III	17	–
18	BSU303	94 y	Survived	V	23	Yes
19	BSU304	1 mo	Died	Ia	23	–
20	BSU305	34 y	Survived	V	1	–
21	BSU306	59 y	Survived	IV	2	–
22	BSU307	79 y	Died	Ia	88	–
23 (*4*)	BSU865, VS	38 y	Survived	II	19	–
	BSU866, BC			–	–	–
24	BSU869	32 y	Died	Ib	8	–
25	BSU870	53 y	Survived	Ia	23	–
Control (*3*)	BSU871	50 y	Survived	Ib	8	Yes

*BC, blood culture; GBS, group B *Streptococcus*; STSS, streptococcal toxic shock syndrome; VS, vaginal swab; –, not found.

We used standard molecular biology techniques for nucleic acid preparation and analysis. We performed molecular typing by multilocus sequence typing (MLST) as described (*5*) and capsular typing by using latex agglutination and PCR serotype determination (*6*) (Table). To analyze the *cov* gene locus, we amplified the genes *covS* and *covR* by PCR (Technical Appendix Table). Resulting PCR products underwent DNA sequencing with internal *cov* primers on an ABI PRISM 310 Genetic Analyzer (Applied Biosystems, Weiterstadt, Germany).

Nucleotide sequence analysis showed that, in 1 of the strains (from patient 18), both genes, the sensor histidine kinase *covS* and the response regulator *covR*, had mutated. In *covR,* at nucleotide position 242, cytosine was replaced by thymine, leading to an amino acid exchange from alanine to valine. In addition, the *covS* gene of this strain showed a 1-bp deletion of adenine at position 895 of the gene, causing a frame shift and leading to a truncated CovS with a stop codon at nucleotide position 926 of the gene. In the control strain that harbored a 3-bp deletion, our previously published finding was confirmed (*3*). In the remaining strains, *cov* alleles matched the gene sequences of completely sequenced GBS strains in the GenBank database (NEM316, 2603V/R, 909A).

During the past few decades, the overall incidence of invasive GBS infections has increased substantially. This trend is particularly noticeable in the elderly and in persons with co-morbid conditions (*7*). Our results regarding age distribution of patients, mortality rate, and frequencies of different GBS serotypes are in line with results of previous studies. Twelve (52%) of 23 patients were >59 years old. The mortality rate for group B STSS (>30%) was similar to that reported for group A STSS (*1*). In a previous case series, which included 13 patients with group B STSS, the mortality rate was 23% (3/13) (*8*). Three-quarter of our strains (19/26 strains) were attributed to serotype V or Ia/Ib. Large epidemiologic studies have frequently implicated serotype s Ia/Ib, III, and V GBS isolates in the etiology of invasive disease in adults (*9*). Apart from sequence type (ST) 1 and ST23 (15/26 strains), the distribution of MLSTs among the GBS isolates was heterogeneous. The highly virulent GBS lineage ST17 was found in only 2 patients (1 adult and 1 child).

The role of *covR/S* mutations in the switch from colonization to invasion has been demonstrated for GAS in a mouse model (*2*). Consistent with these findings, GAS strains isolated from STSS patients frequently carry mutations in this operon (*10*). Similarly, a 3-bp deletion in the *covR* gene was detected in a GBS strain that caused STSS and necrotizing fasciitis (*3*). However, our investigations on a larger collection of GBS isolates did not confirm a high *cov* mutation rate. Only 1 of 25 GBS clones demonstrated a mutation. From 1 patient, a colonizing and invasive isolate (same clone) was available (*4*); that isolate showed no mutation in *covR/S*.

Our results should be interpreted with caution because the absolute number of included patients is small, and the cases were pooled from various centers. Nonetheless, Ikebe et al. found *covR/S* mutations in 76 (46.3%) of 164 GAS strains causing STSS and in only 1.7% of 59 strains without invasive disease (*10*). In light of these results, the low frequency of mutations found in our collection is surprising. Yet, the association with *covR/S* mutations and GBS TSS in a case report has been shown previously and confirmed here. However, GBS harbors multiple 2-component systems and stand-alone regulators. Our findings indicate that different virulence regulators may be involved in the pathogenesis of fulminant GBS disease.

Technical Appendix Primers used in study of group B streptococcal toxic shock syndrome and *covS/R* mutations.
