# Supplementary material for: Group B Streptococcal Toxic Shock Syndrome and covR/S Mutations Revisited
Source: Emerg Infect Dis. 2017 Jan;23(1):150–2. doi: 10.3201/eid2301.161063 (PMC5176209; doi:10.3201/eid2301.161063)
Supplement: Technical Appendix — Primers used in study of group B streptococcal toxic shock syndrome and covS/R mutations. [file 16-1063-Techapp-s1.pdf]

Article DOI: http://dx.doi.org/10.3201/eid2301.161063

Group B Streptococcal Toxic Shock Syndrome and *covS/R* Mutations Revisited

Technical Appendix

Technical Appendix Table. Primers used in study of group B streptococcal toxic shock syndrome and covS/R mutations

Primer name	Nucleotide sequence
covSR_fw	5′-GCGCCTCTCTTATCACCTC-3′
covSR_rev	5′-GAAGGTTGGTGTAGATGGG-3′
covSR_fw2	5′-TAAGATCATGAGAGCTTGCT-3′
covSR_rev2	5′-GTAGTTTCCGTGATTTGTCA-3′
covSR_fw3	5′-CGGACCTTACTTCTAATTTC-3′
covSR_rev3	5′-CTCTAGTTTGACATCCGATG-3′
covSR_rev_1.2	5′-GTCTCGTTAGAACTACTACA-3′
covSR_fw_5′ende	5′-GTGATTTCGAAACCATCCAT-3′
covSR_rev_5′ende	5′-GTGAACTAAATCAAGGGACT-3′
